# New insights on commemoration of the dead through mortuary and architectural use of pigments at Neolithic Çatalhöyük, Turkey

**DOI:** 10.1038/s41598-022-07284-3

**Published:** 2022-03-08

**Authors:** E. M. J. Schotsmans, G. Busacca, S. C. Lin, M. Vasić, A. M. Lingle, R. Veropoulidou, C. Mazzucato, B. Tibbetts, S. D. Haddow, M. Somel, F. Toksoy-Köksal, C. J. Knüsel, M. Milella

**Affiliations:** 1 Laboratoire d’Anthropologie des Populations Passées et Présentes (PACEA), UMR 5199, Université de Bordeaux, Pessac, France; 2 Centre for Archaeological Science, University of Wollongong, Wollongong, Australia; 3 Palermo, Italy; 4 ARC Centre of Excellence for Australian Biodiversity and Heritage, University of Wollongong, Wollongong, Australia; 5 Berlin, Germany; 6 School of History, Archaeology, and Religion, Cardiff University, Cardiff, UK; 7 Museum of Byzantine Culture, Hellenic Ministry of Culture and Sports, Thessaloníki, Greece; 8 Department of Anthropology, Stanford University, Stanford, USA; 9 Department of Archaeology, University of Exeter, Exeter, UK; 10 Department of Cross-Cultural and Regional Studies, University of Copenhagen, Copenhagen, Denmark; 11 Department of Biological Sciences, Middle East Technical University (METU), Ankara, Turkey; 12 Department of Geological Engineering, Middle East Technical University (METU), Ankara, Turkey; 13 Department of Physical Anthropology, Institute of Forensic Medicine, University of Bern, Bern, Switzerland

**Keywords:** Anthropology, Archaeology, Cultural evolution

## Abstract

The cultural use of pigments in human societies is associated with ritual activities and the creation of social memory. Neolithic Çatalhöyük (Turkey, 7100–5950 cal BC) provides a unique case study for the exploration of links between pigments in burials, demographic data and colourants in contemporary architectural contexts. This study presents the first combined analysis of funerary and architectural evidence of pigment use in Neolithic Anatolia and discusses the possible social processes underlying the observed statistical patterns. Results reveal that pigments were either applied directly to the deceased or included in the grave as a burial association. The most commonly used pigment was red ochre. Cinnabar was mainly applied to males and blue/green pigment was associated with females. A correlation was found between the number of buried individuals and the number of painted layers in the buildings. Mortuary practices seem to have followed specific selection processes independent of sex and age-at-death of the deceased. This study offers new insights about the social factors involved in pigment use in this community, and contributes to the interpretation of funerary practices in Neolithic Anatolia. Specifically, it suggests that visual expression, ritual performance and symbolic associations were elements of shared long-term socio-cultural practices.

## Introduction

The use of pigments by past societies has been considered as evidence for modern human behaviour based on its association with symbolic activities and rituals^1–4^. Middle Stone Age sites younger than 180 kya in Africa^3,5–9^ and the Middle East^10–12^ show systematic evidence of red pigment use. In mortuary contexts, pigments became prevalent during the Upper Palaeolithic in Europe^13–17^, but in the Upper Palaeolithic Near-East, the number of recovered burials remains relatively small^18,19^, as is the evidence for ochre processing and ochre use^18–21^.

The earliest Near Eastern case of pigment use in an architectural context dates back to the Early Natufian period with a building coated with plaster and red paint from the site of Ain Mallaha-Eynan^22^. Also attributed to Natufian traditions is the origin of complex manipulations of skeletal elements such as the removal, modelling and painting of crania^23,24^, although evidence for funerary pigments remains limited to only a few sites; Azraq 18^24^, Ain Mallaha-Eynan^25^ and Hayonim cave^26^. In the early Pre-Pottery Neolithic period (PPNA) both architectural and funerary evidence of pigments continues to remain scarce. The sites of Dja’de el-Mughara and Mureybet, located along the Syrian Euphrates (northern Levant), show evidence of architectural paintings during different Pre-Pottery Neolithic phases with emerging variation in mortuary practices, limited pigment applications and secondary deposits in clear connection with the architecture^25,27–31^. It is only during the second half of the 9^th^ millennium and the 8^th^ millennium BC that architectural paintings and funerary pigment use seem to spread across a large area reaching from the Southern Levant to Central Anatolia^25,32^. Increasingly common examples of secondary handling of human remains in single or collective burials, removal of crania and the presence of elaborate burial associations signal selection and increased symbolism that continued through the Neolithic^24,33–36^.

Focussing on Anatolia specifically, changes in mortuary practices were accompanied by a growing variability in pigment processing and application. The Epipalaeolithic levels of Pınarbaşı show evidence of ochre processing as well as the presence of colourants in a burial context^37,38^. The Early Neolithic site of Göbekli Tepe, known for its architectural decorations of stone carvings and bas reliefs, provided cranial fragments with traces of red ochre but was lacking burials^39^. Near the northern extension of the Fertile Crescent, black and red pigments were observed on bones recovered at the Early Neolithic sites of Körtik Tepe, Demirköy and Hasankeyf Höyük^40–42^. Several phases from Çayönü revealed buildings with red paint and ‘skull buildings’, but without reported pigment on human remains^43,44^. In Central Anatolia, red paint was found on walls, floors and benches in ‘special’ or communal buildings at Aşıklı Höyük^45^. In contrast, ochre in burials and on ornaments was rare and traces were only found on one bead, one shell and in the burials of two children and one adult female^46–48^. At the site of Musular, red pigment was observed on floors and benches, but not reported on human remains^49^. In Boncuklu Höyük, red colourants were attested on walls and floors, as well as in burials; occasionally on crania and with secondary/tertiary depositions of human remains^50–52^. The late Neolithic phases of Köşk Höyük revealed a wall painting^32,53^ and 13 out of 19 plastered human crania were covered with red ochre^54,55^. The site of Tepecik-Çiftlik has one architectural feature with red and blue colourants in a building dated to the final Neolithic period^32^, but no pigments were reported on any of the more than 170 human remains^56,57^.

These examples suggest important symbolic, ritual and social behaviour in the Anatolian Epipalaeolithic and Neolithic periods. Repetitive use of imagery, secondary mortuary treatments and increased circulation of human bones highlight integrated systems of social memory by linking the living to the dead and, thus, creating the basis for social differentiation^58–60^. A problem raised in the past, however, is that many discussions focus on post-mortem skull removal while other factors central to mortuary practices remain under-explored, such as associations between burial contexts and architectural features^61^. The fact that archaeological contexts of paintings and burials are not physically connected is often a reason to ignore correlations between the two, despite ethnographic examples demonstrating the overlap between domestic and ritual practice^60,62–67^. The lack of analyses linking the dead, the physical space of the house and colourant use has been raised about Çatalhöyük specifically^62,68,69^, but no attempt has been made so far to fill this gap. The long-running, 25-year excavation project at Çatalhöyük provides the opportunity to compare the use of colourants in architectural and funerary contexts in several buildings from successive periods in order to discuss the possible social relevance and symbolic meaning of pigments within this Neolithic society.

Çatalhöyük is one of the largest Neolithic settlements in Anatolia^35,70^. Archaeological excavations of the site took place between 1961 and 1965 under the direction of James Mellaart, and from 1993 to 2017 by Ian Hodder^71^. The Neolithic occupation on the East Mound at Çatalhöyük can be placed between 7100 cal BC and 5950 cal BC^72^, a timeframe encompassing the Late Pre-Pottery Neolithic B and the beginning of the Pottery Neolithic.

The Neolithic occupation of Çatalhöyük is subdivided into four phases according to stratigraphic, radiocarbon, archaeological and anthropological data^72–75^. These phases pinpoint important cultural and demographic changes. Between the Early (7100–6700 cal BC) and the Middle occupation periods (6700–6500 cal BC) the population gradually rose, houses became larger and symbolic elaboration at the site increased^74,75^. During the Late (6500–6300 cal BC) and Final (6300–5950 cal BC) occupation periods households became more autonomous^75,76^. From 6500 cal BC until the abandonment of the East Mound available data suggest greater mobility of the population, economic independence among households, and increased specialisation and differentiation^74,75^. The same traits are shared with the later cultural and socio-economic development during the Chalcolithic between the East Mound and neighbouring West Mound^74–76^.

The excavation of Neolithic Çatalhöyük has produced an important number of primary and secondary depositions. Burials mostly occurred within domestic structures during the occupation phase of houses and for a minority in construction and abandonment phases of the buildings^77^. Adults were most often placed in a flexed position, located beneath the northern and eastern platforms of the central room. Perinate, neonate and infants were buried in more variable locations within the house^77–80^. In the Final period, the houses no longer accommodate distinct platforms with burials^74^ and there is an increase in the ratio of secondary to primary depositions^77^. A well-known feature at Çatalhöyük is the presence of pigments in both funerary and architectural contexts. Although the use of pigments is recognised as one of the defining elements of this community, previous research has focused mainly on one aspect (presence in architectural contexts) of its variability^32,68,81–83^.

This study provides, for the first time, a fine-grained quantitative analysis of the available funerary and architectural data related to pigment use at Neolithic Çatalhöyük. By means of a joint study of different datasets, until recently considered in isolation from each other, the aim is to offer a new perspectives on the possible social processes involved in the use of pigments in this society. To do this, the following research questions are addressed: (a) Which pigments were used at Neolithic Çatalhöyük and in which relative frequency? (b) In which manner were pigments deposited in funerary contexts? (c) What are the associations between pigment use, type of deposition, and sex and age-at-death of the deceased? (d) Is there a chronological variability in the association between architectural use of colourants and mortuary patterns?

## Results

### Variability in pigment use at Çatalhöyük

The most frequent pigment encountered at Çatalhöyük is red ochre, known as haematite or iron oxide (Fe_2_O_3_). It present in different depositional contexts across the site including burials and as part of architectural decoration of walls and installations within houses, but also on figurines, clay balls and surviving wooden posts^32,84–89^. Less common pigments include yellow ochre, cinnabar, blue azurite and green malachite. Yellow ochre, or goethite (FeO(OH)), was found in certain wall paintings from the earlier occupation levels and in some burials^81^. Cinnabar (HgS) or vermilion, a scarlet red form of mercury, was mainly found on human remains, shells and occasionally mixed with ochre for wall paintings^81,85^ (see Supplementary Fig. S1). Malachite (Cu_2_CO_3_(OH)_2_) and azurite (Cu_3_(CO_3_)_2_(OH)_2_) were uniquely present as burial associations^81,85,90^. The latter came from at least two different sources. The dark blue variant contained arsenic, antimony, lead and zinc derived from an arsenic rich geology, as observed in the area around Niğde, close to the Black Sea, or in the Kutahya region of western Anatolia. These elements were not present in the light blue version of azurite^85^ (see Supplementary Figs. S2 and S3).

### Funerary pigment use

11% of all primary and secondary depositions (62 out of 567) shows evidence of pigment inclusion, either directly on the skeletal remains or indirectly as burial association (henceforth defined as movable items associated with the grave and the deposited individual, regardless of intent^91^) (Table 1).

**Table 1:** List of Çatalhöyük primary and secondary human remains with pigments classified as (a) skeletons with direct pigment traces and (b) individuals with burial associations that contained pigment.

	Period	Building	Skeleton (unit) number	Deposition category	Age-at-death	Sex	Orientation of the cephalic extremity	Side	Position	Single or collective burial	Colour	Pigment details	Presence of phytoliths
**(a)**													
1	Late	150	32818	Primary disturbed	MA	M	E	Supine	Flexed	Collective	Red	Cinnabar as stripe on frontal bone cranium	No evidence
2	Late	150	23972	Secondary	YA	M	Cranium only	Cranium only	Cranium only	Collective	Red	Red pigment on the cranium	No evidence
3	Late	42	11330	sEcondary	OA	F?	Cranium only	Cranium only	Cranium only	Collective	Red	Iron-oxide on plastered cranium	No evidence
4	Late	108	17533	Primary disturbed	MA	M	W	Right	Flexed	Collective	Red	Cinnabar on left side of the cranium	No evidence
5	Middle	132	32741	Primary	MA	indet	N	Left	Flexed	Collective	Red	Iron-oxide on occipital fragments cranium and on ribs	Yes
6	Middle	132	32762	Primary	OA	M?	SW	Prone	Flexed	Single	Red	Iron-oxide on the cranium, right ulna, vertebral column and right femur	No evidence
7	Middle	132	32045	Primary	Child (8–12 years)	tytd	W	Left	Flexed	Single	Red	Iron-oxide on parietal bone cranium	Yes
8	Middle	5	22196	Secondary	YA	M	Cranium only	Cranium only	Cranium only	Collective	Red	Cinnabar as stripe on frontal bone cranium covered with phytoliths	Yes
9	Middle	132	32770	Primary disturbed	YA	M	W	Left	Flexed	Collective	Red	Red pigment on cranium and parts of the infracranial skeleton	Yes
10	Middle	131	32330	Primary disturbed	Adolescent (15–20 years)	indet	Cranium only	Cranium only	Cranium only	Single	Red	Cinnabar on cranium	Yes
11	Middle	52	30523	Primary disturbed	Infant (18-24 months)	tytd	W	Left	Flexed	Collective	Red	Cinnabar on frontal bone cranium	No evidence
12	Middle	167	23805	Primary	Infant (1–2 years)	tytd	S	Right	Flexed	Single	Red	Cinnabar on frontal bone cranium	Yes
13	Middle	1	1912	Primary	Infant (1–2 years)	tytd	E	Left	Flexed	Collective	Red	Red pigment concentrated around the right hand bones	Yes
14	Middle	114	30007	Primary disturbed	YA	M	E	Right	Flexed	Collective	Red	Cinnabar on cranium and specks on cervical vertebrae	Yes
15	Middle	114	30010	Primary disturbed	Infant	tytd	S	Left	Flexed	Collective	Red	Cinnabar on cranium	Yes
16	Middle	77	20685	Primary disturbed	Adult	M?	SW	Left	Flexed	Single	Red	Cinnabar on temporal bone cranium	Yes
17	Middle	114	8598	Primary	YA	M	E	Right	Flexed	Collective	Red	Iron-oxide on infracranial skeleton	No evidence
18	Middle	50	10840	Primary	MA	M	W	Right	Flexed	Collective	Red	Red pigment on whole skeleton with concentration around the thorax	No evidence
19	Early	6	4406	Primary	Infant (6 months-1 year)	tytd	W	Prone	Flexed	Single	Red	Red pigment on the lower part of the skeleton and lower limbs	Yes
20	Early	6	4615	Primary	OA	F	S	Right	Flexed	Single	Red	Red pigments on whole skeleton with concentration around the thorax	No evidence
21	Early	43	22335	Primary	Infant (18-24 months)	tytd	NE	Supine	Flexed	Collective	Red	Cinnabar and iron-oxide on frontal bone cranium	No evidence
22	Early	6	4424	Primary	Infant (1–2 years)	tytd	N	Left	Flexed	Single	Red	Cinnabar on frontal bone cranium	Yes
23	Early	6	4458	Primary	Infant (8 months)	tytd	?	Supine	Flexed	Single	Red	Red pigments on whole skeleton	Yes
24	Early	17	21884	Primary disturbed	YA	F	W	Right	Flexed	Single	Red	Iron-oxide on whole skeleton with more staining on the left side	Yes
25	Early	17	23238	Primary disturbed	Child (3 years)	tytd	N	Left	Flexed	Collective	Red	Iron-oxide on pelvis and lower limbs and below the skeleton	No evidence
26	Early	17	21817	Primary	MA	F?	W	Left	Flexed	Single	Red	Iron-oxide on whole skeleton	Yes
27	Early	17	21855	Primary	Child (2–3 years)	tytd	E	Left	Flexed	Single	Red	Iron-oxide concentrated below torso and around the upper limbs	Yes
28	Early	17	22522	Primary	MA	F?	W	Right	Flexed	Single	Red	Cinnabar on cranium and iron-oxide on infracranial skeleton, more concentrated on left side	Yes
29	Early	17	5177	Primary	Child (2–3 years)	tytd	W	Right	Flexed	Single	Red	Cinnabar on temporal bone cranium	Yes
30	Early	17	23236	Primary	OA	M?	E	Supine	Flexed	Single	Red	Red pigment on infracranial skeleton	Yes
31	Early	161	32645	Primary	OA	M?	N	Left	Flexed	Collective	Red	Iron-oxide on whole skeleton, more concentrated on the right side	Yes
32	Early	161	32646	Primary	OA	M	W	Right	Flexed	Collective	Red	Iron-oxide on whole skeleton	Yes
33	Early	160	32437	Primary	OA	M	W	Right	Flexed	Single	Red	Cinnabar on cranium and iron-oxide on infracranial skeleton, more concentrated on left side	Yes
34	Early	23	4853	Primary	Prenate (30–32 weeks)	tytd	E	Right	Flexed	Collective	Red	Red pigment on cranium	Yes
35	Early	23	4861	Primary	Prenate (38–38 weeks)	tytd	?	Right	Flexed	Collective	Red	Red pigment on cranium	Yes
36	Early		23237	Primary	Child (3 years)	tytd	W	Left	Flexed	Single	Red	Iron-oxide on whole skeleton	No evidence
**(b)**													
1	Late	60	13162	Primary disturbed	YA	F	W	Right	Flexed	Collective	Green	Malachite as pigment lump with bone applicator	No evidence
2	Late	129	19460	Primary	Child (10–14 years)	tytd	W	Left	Flexed	Collective	Red and blue	Red and blue pigment on one of two mirrors present as burial association	No evidence
3	Late	150	32818	Primary disturbed	MA	M	E	Supine	Flexed	Collective	Red	Shell with cinnabar	No evidence
4	Late	150	Several individuals	Primary disturbed						Collective	Red and blue	Azurite and red pigment as lumps	no evidence
5	Middle	97	19224	Primary	YA	F	SW	Left	Flexed	Collective	Red	Red pigment on phytoliths	Yes
6	Middle	50	2842	Primary disturbed	Infant (2 months—3 years)	tytd	E	Right	Flexed	Collective	Red	Shell with cinnabar	Yes
7	Middle	5	22196	Secondary	YA	M	Cranium only	Cranium only	Cranium only	Collective	Red	Shell with cinnabar	Yes
8	Middle	131	23126	Primary	Adolescent (16–20 years)	tytd	W	Left	Flexed	Collective	Red and Blue	Iron-oxide and azurite as lumps. Iron-oxide present on a mirror as burial association	Yes
9	Middle	131	31705	Primary	Adolescent (16 years)	F	N	Left	Flexed	Collective	Blue	Azurite on wooden bowl	Yes
10	Middle		21672	Primary	OA	F	E	Right	Flexed	Single	Green	Malachite on two bone applicators	Yes
11	Middle	52	20655	Primary	MA	F	W	Left	Flexed	Single	Red	Shell with red pigment	Yes
12	Middle	49	17939	Primary	Infant (1–3 years)	tytd	S	?	Flexed	Single	Red	Shell with cinnabar	Yes
13	Middle	131	23075	Primary	Neonate	tytd	W	Left	Flexed	Single	Red	Red pigment on wooden bowl	Yes
14	Middle	52	30514	Primary	MA	M?	W	Supine	Flexed	Collective	Red	Shell with red pigment	No evidence
15	Middle	77	19500	Secondary	OA	M	Cranium only	Cranium only	Cranium only	Single	Red	Red pigment on schist palette	No evidence
16	Middle	77	30199	Primary disturbed	Infant	tytd	NW	Right	Flexed	Single	Red	Shell with cinnabar	Yes
17	Middle	3	8184	Primary	Infant	tytd	W	Left	Flexed	Collective	Red and Green	Shell with red pigment and malachite with bone applicator	Yes
18	Middle	49	17457	Primary	Infant	tytd	?	Left	Flexed	Collective	Red and blue	One shell with cinnabar, one shell with ochre, cinnabar staining on phytoliths from basket and lump of blue pigment with bone applicator	Yes
19	Middle	3	8115	Primary	MA	F	E	Supine	Flexed	Collective	Blue	Lump of blue pigment	No evidence
20	Middle		16309	Primary	OA	F	W	Right	Flexed	Collective	Green	Lump of malachite	No evidence
21	Middle		16308	Primary	OA	F	W	Right	Flexed	Collective	Blue	Lump of azurite and bone applicator	Yes
22	Middle	1	2105	Primary	Infant	tytd	N	Supine	Flexed	Collective	Blue	Lump of azurite	No evidence
23	Middle	132	21685	Primary	OA	F?	W	Right	Flexed	Single	Red	Shell with red pigment	Yes
24	Early	17	22516	Primary	Child (10 years)	tytd	W	Left	Flexed	Single	Red	Lump of cinnabar	Yes
25	Early	43	10529	Primary	Child (10–12 years)	tytd	E	Prone	Flexed	Single	Red	Lump of red pigment	No evidence
26	Early	23	4853	Primary	Prenate (30-32 weeks)	tytd	E	Right	Flexed	Collective	Red	Basket stained with red pigment	Yes

*OA* old adults, *MA* middle adults, *YA* young adults, *F* female, *F?* possible female, *indet* indeterminate, *M?* possible male, *M* male, *tytd* too young to determine, *N* North, *E* East, *S* South, *W* West. Unidentified pigment is mentioned as colour. Table generated with Microsoft Office Excel 2016 (https://www.microsoft.com/en-us/microsoft-365/excel).

**Figure 1:**
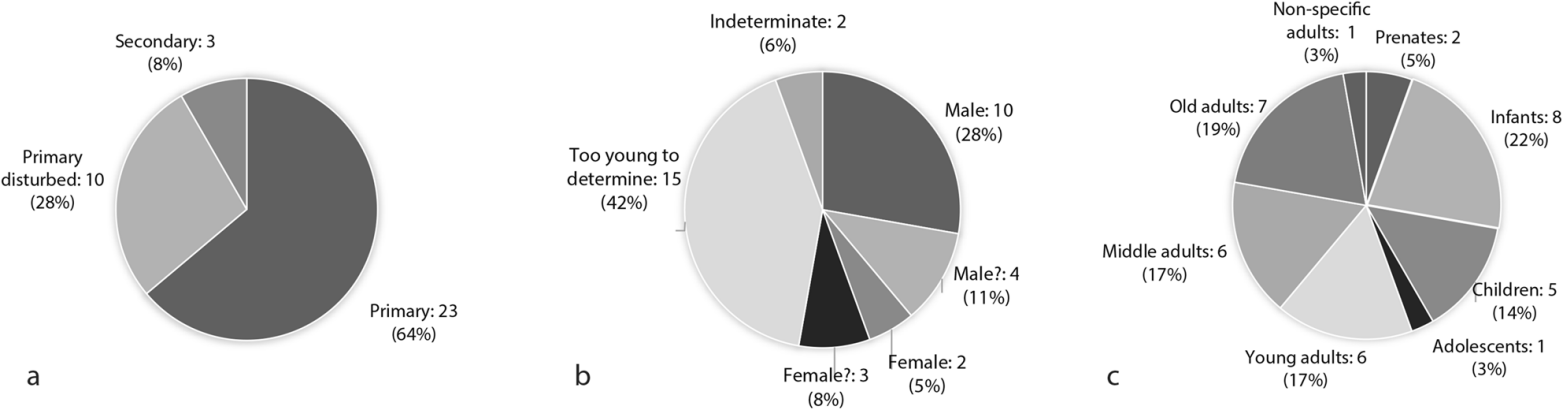
Distribution of Çatalhöyük human remains with direct pigment traces by (**a**) deposition category, (**b**) sex and (**c**) age-at-death. Charts generated with Microsoft Office Excel 2016 (https://www.microsoft.com/en-us/microsoft-365/excel).

Traces of pigment found directly on skeletal remains are always red in colour, consisting of either ochre or cinnabar. Pigment was present on 36 individuals (6.3% of all primary and secondary depositions) (Table 1). Of these, 33 (92%) were primary depositions and three (8%) were secondary depositions (Fig. 1a). There were more males (39%) with direct pigment traces than females (13%) (Fig. 1b). The proportion of skeletons receiving direct pigment treatment is slightly smaller for younger individuals (44%) than for adults (56%) (Fig. 1c). Some 67% of the skeletons with direct pigment traces show evidence of some sort of funerary containment based on the presence of phytoliths, in the form of rope, matting or basketry.

The majority of individuals with direct pigment traces display pigment on the cranium (n = 30; 83%). Of these, 18 are adults and 12 are subadults. Among these cases, sex was determined for 17 individuals (12 males and 5 females). The 30 individuals with pigment on the cranium were further separated into those with pigment exclusively on the cranium and those who displayed pigment staining on both cranial and infracranial elements. The occurrence of these two forms of pigment applications differ significantly between adults and subadults (*p* = 0.011) with the application of pigment exclusively on the cranium being more frequent in subadults.

**Figure 2:**
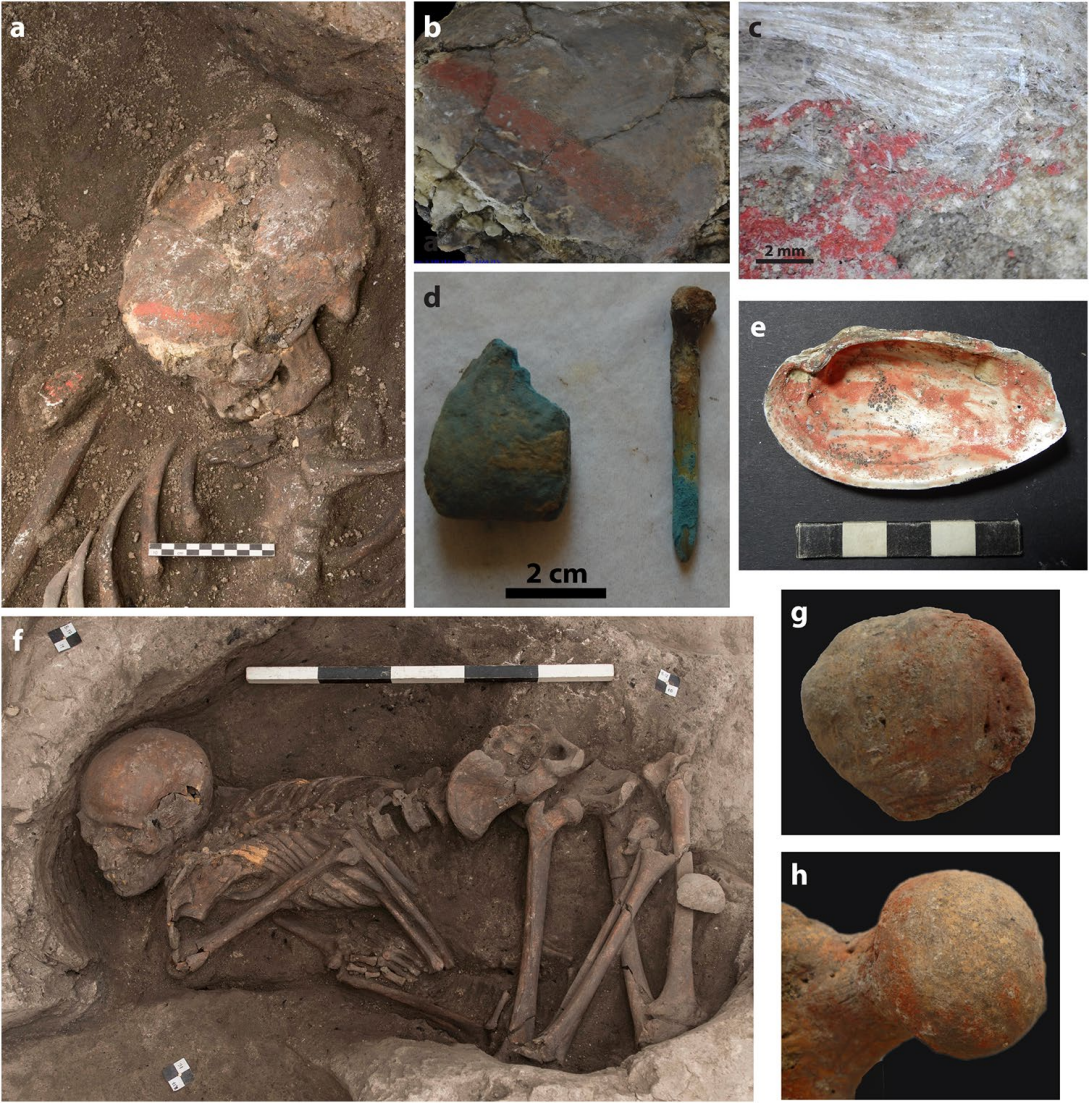
Examples of funerary pigment use at Çatalhöyük. (**a**) In situ photograph from skeleton 32818 with cinnabar stripe and a shell with cinnabar deposited at the right shoulder (Photograph by J. Quinlan); (**b**) Detail of the cinnabar stripe (Photograph by M. Milella); (**c**) Microscopic image of the frontal bone of skeleton 22196 showing a cinnabar layer with unstained phytoliths on top (Photograph by E. Schotsmans); (**d**) Bone ‘applicator’ with lump of blue pigment recovered with skeleton 16308 (Photograph by J. Quinlan); (**e**) *Unio* shell ‘palette’ with cinnabar (22194.X6) (Photograph by R. Veropoulidou); (**f**) Individual 21884 was buried on its right side with the skeletal elements on the uppermost and left side of the skeleton more intensely stained with red pigment (Photograph by J. Quinlan); (**g**) Right patella of skeleton 21884 was more stained on its medial side (Photograph by E. Schotsmans); (**h**) The partial discolouration of the left femoral head confirms that individual 21884 was flexed and fleshed when the ochre was applied, leaving the main part of the femoral head unstained (Photography by E. Schotsmans). Figure generated with Adobe illustrator 23.0.6 (http://www.adobe.com/fr/products/illustrator.html).

With regard to the anatomical distribution of different types of pigments, iron oxide is observed on the cranium and/or on the infracranial skeleton. In contrast, cinnabar only occurs on the cranium of 14 individuals (2.5% of the total sample or 39% of the skeletons with direct pigment traces), mostly from primary contexts (13 out of 14), including both subadults (n = 7) and adults (n = 7), the latter of whom are mostly males (M = 5, M? = 1, F? = 1). The presence of cinnabar is restricted to the frontal and/or temporal bones of the cranial vault. In two individuals (skeleton 32818 and 22196) cinnabar staining takes the form of a stripe (Fig. 2a,b). In two other cases, phytoliths were found adhering to the cinnabar (skeletons 5177 and 22196) (Fig. 2c).

**Figure 3:**
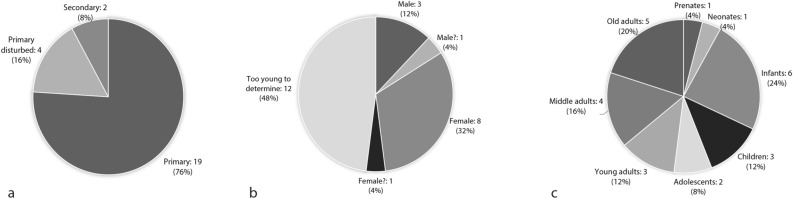
Distribution of Çatalhöyük human remains with pigments as burial association by (**a**) deposition category, (**b**) sex and (**c**) age-at-death. Charts generated with Microsoft Office Excel 2016 (https://www.microsoft.com/en-us/microsoft-365/excel).

Burial associations with pigments were present as red, blue or green pigment in 25 burials (4.6% of all primary and secondary depositions) from mainly primary depositions either as lumps or as stained objects (Fig. 3a, Table 1). The data show a relatively even distribution between adults (48%) and subadults (52%) (Fig. 3c) with more females (36%) than males (16%) having pigment as a burial association (Fig. 3b). Pigments were associated with six bone objects and present on eleven stained shells, on phytoliths, probably from matting, on two baskets, two wooden bowls, one schist palette and on two mirrors (Table 1). Blue and green pigments were associated only with adult females (n = 5) and subadults (n = 6).

**Figure 4:**
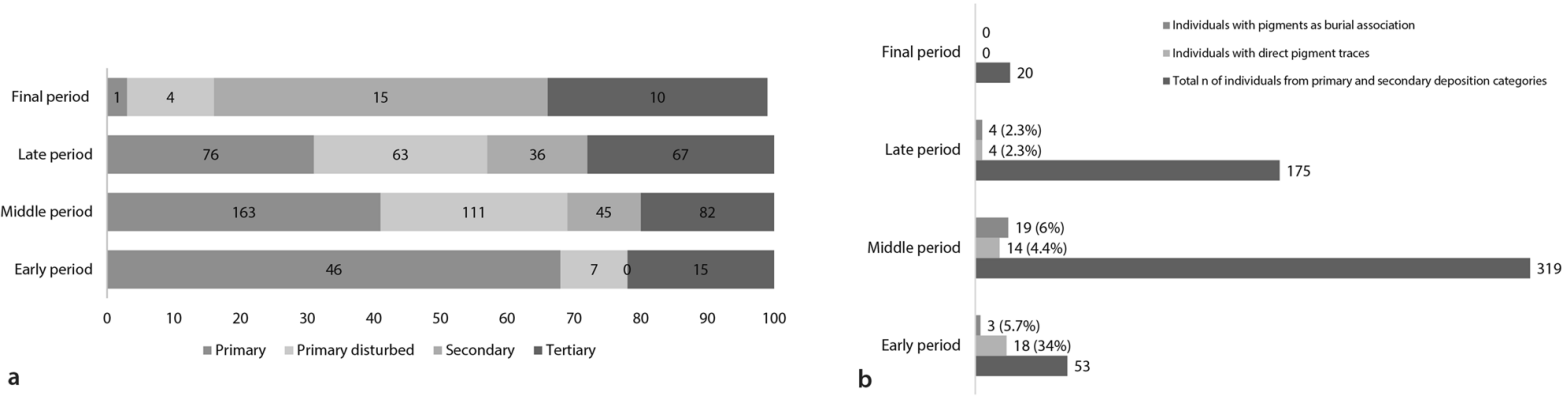
(**a**) Diachronic distribution of the different depositional categories; (**b**) Number of individuals and relative percentages of individuals with direct pigment traces and with pigments as associated items by occupation period (n = number). Charts generated with Microsoft Office Excel 2016 (https://www.microsoft.com/en-us/microsoft-365/excel).

Diachronically, the relative frequency of primary burials decreases over time while the proportions of secondary and tertiary depositions increase. This trend is most clearly visible in the secondary deposition category, being absent in the Early occupation period but becoming the largest category in the Final occupation period (Fig. 4a)^77^. Percentages of individuals with pigments from the Early, Middle, Late, and Final occupation periods amount to 39.7%, 10.4%, 4.6%, and 0%, respectively (Fig. 4b).

The frequency of the two studied pigment categories, those found as direct applications or as burial associations, changed significantly over time (*p* = 0.004). The occurrence of pigments in burials from the Early occupation period consisted almost entirely of directly applied colourants. In the Middle and Late periods the occurrences of pigments on human remains and as burial association became more even. An additional observation is that a combination of cinnabar on the cranium and ochre on the infracranial skeleton is observed only in the Early occupation period (n = 3). Two individuals had traces of applied pigment as well as a burial association (skeletons 4853 and 32818). Four subadults from the Middle (n = 3) and Late (n = 1) period were found with both red and blue/green pigments as burial association (Table 1).

### Architectural use of colourants and its associations with burials

**Figure 5:**
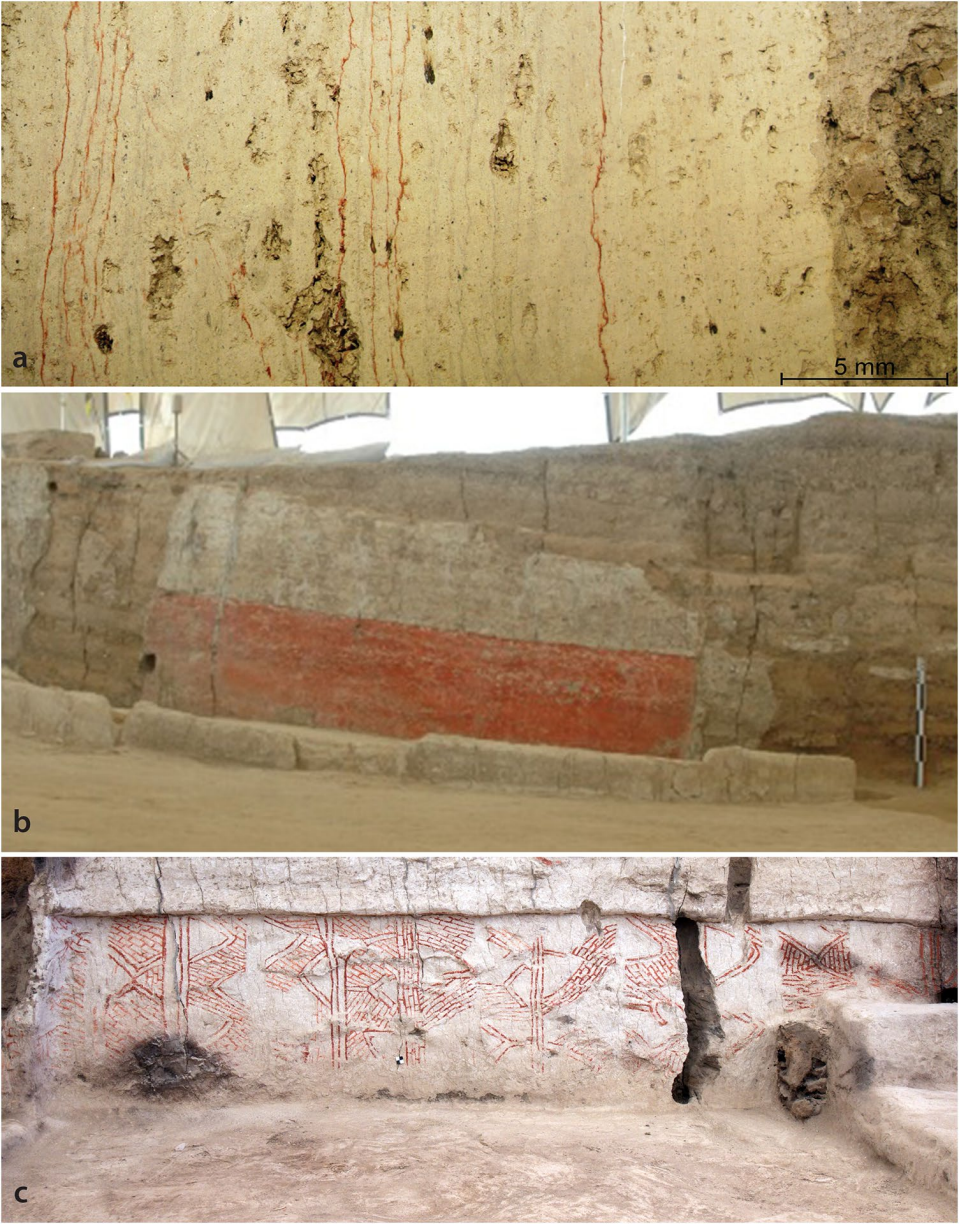
(**a**) Microscopic image of a multi-layered plaster from building 17, the consecutive marl layers are observable, separated by red pigment or by soot (Photograph by G. Busacca); (**b**) Example of a monochromatic red wall painting from building 59 (Photograph by J. Quinlan); (**c**) Example of a wall painting with geometric motif from building 80 (Photograph by J. Quinlan). Figure generated with Adobe illustrator 23.0.6 (http://www.adobe.com/fr/products/illustrator.html).

A total of 178 archaeological units with 323 painted layers (Fig. 5a) occurred on a variety of plaster-lined house interior features, such as walls (42.3%), platforms (24.7%), niches (6.6%), benches (6.6%), post/pillars (6.6%), floors (4.9%) and other (8.3%). With regard to painted designs, the vast majority of the studied architectural use of colourants was composed of monochromatic red layers (58.6%) (Fig. 5b), followed by a portion with motifs that could not be identified due to poor preservation or insufficient exposure (23.1%). Geometric motifs made up about 15% of the painting corpus (Fig. 5c), while hand motifs (2.1%) and combinations of geometric designs and hand motifs (0.6%) were less frequent.

From a diachronic perspective, all investigated buildings from the Early and Middle occupation levels showed at least some evidence of architectural colourant use (62% and 71%, respectively), although substantial variability exists in the extent of elaboration. In contrast, in the Late occupation levels, a significantly lower proportion of buildings yielded evidence of paintings (32%) (*p* = 0.018).

The spatial distribution of colourant use within the houses also showed period-specific patterns^92^. During the Early period, they mainly appear clustered in the eastern and northeastern parts of the house. During the Middle occupation period, the vast majority of paintings were located where burials occurred in the ‘clean’ areas of houses (‘dirty’ areas being linked with hearths, ovens and cooking^93^), and associated with interior features such as white-plastered platforms, benches, plaster or bone embellished installations and intramural burials. In the Late period, architectural uses became less frequent and spread to all walls of the houses.

There is a wide range of variation in the number of buried individuals contained within the 66 studied buildings, ranging between 0 and 64. Among the buildings that do contain burials (54 out of 66), the proportion of burials that have pigments, either through direct application or as burial associations, also varies considerably (0–38%). Focussing on the more fully excavated buildings (i.e., at least 75% excavated; 23 buildings with a total of 398 buried individuals), 87% contain coloured wall layers (20 out of 23), and over 94% of the recovered individuals, buried either with or without pigments (37 and 339 cases respectively), originated from the buildings with painted wall layers. The remaining 22 buried individuals, which all show no sign of pigment use, were found in two buildings that do not contain any painted layers. Overall, across all the buildings, there is a correlation between the number of buried individuals without pigments and the number of buried individuals with pigments (z = 2.21, *p* = 0.02). This means that when the number of burials without pigment increases in a building, the number of burials with pigments tend to increase. In terms of the depositional context of the recovered burials, there is also a correlation between the number of primary burials (including those that were disturbed) and the number of tertiary depositions (z = 2.63, *p* = 0.009).

**Figure 6:**
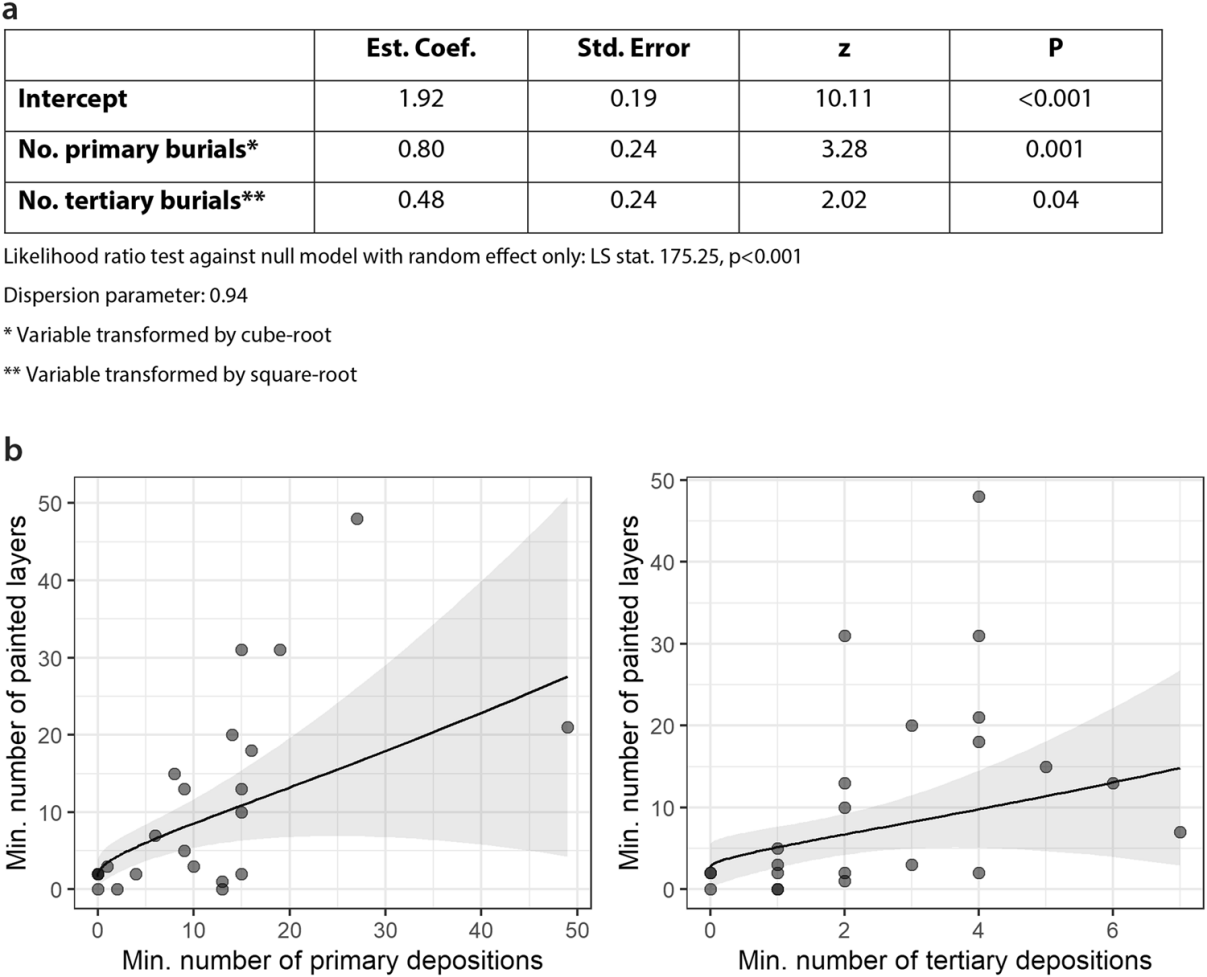
(**a**) Summary of the linear model and the modelled relationships; (**b**) The relationship between primary (left panel)/tertiary (right panel) depositional contexts and the minimum number of painted wall layers. The black solid lines depict the modelled effect of primary and tertiary depositions on the painted layers; the grey area represents the 95% confidence interval of the mean predicted values. Table and graphs generated with R × 64 v4.1.0 (https://cran.r-project.org/bin/windows/base/old/4.1.0/) and combined in Adobe illustrator 23.0.6 (http://www.adobe.com/fr/products/illustrator.html).

To understand the relationship between the number of buried individuals, irrespective of pigment presence, and the number of painted layers among the studied buildings, a generalised linear model (GLM) was built with a negative binomial error distribution (dispersion parameter = 0.94). The variance inflation factor of the two predictor variables is low (1.23 for both primary and tertiary depositions), suggesting that the model is not affected by collinearity. Figure 6a summarises the linear model and the modelled relationships. The results indicate that both primary and tertiary burial counts have a positive relationship with the number of painted layers. This suggests that the quantity of painted wall layers among the buildings is partly connected to the number of primary burials and partly to the number of tertiary depositions (Fig. 6b). Importantly, the relationships between these two variables in association with the number of painted layers are statistically independent from each other, meaning that the number of painted layers tends to increase when the number of either primary *or* tertiary burials increases. This finding implies that mortuary rites accompanying primary burials and tertiary depositions may have been separate, but both in association with the occurrence of painted layers. It should be noted that tertiary depositions were recovered from occupation as well as construction, modification and closure/abandonment layers, which might explain the weaker relationship between tertiary depositions and painted layers. This means that the association between architectural use of colourants and skeletal remains from tertiary contexts may be indirect, rather than directly associated with the occupation of the house alone, hence confirming that different mortuary rites might be in place for the later phases of the sequential funerary practices.

## Discussion

The results hint at substantial variability in mortuary treatment for certain members of the community. The inhabitants of Çatalhöyük practiced funerary differentiation which is clear when considering that only a small number of individuals were treated with pigments. Whereas the choice to use pigments or not, as part of funerary treatment, seems not to have been influenced by the sex and age of the deceased, the choice of specific pigments such as cinnabar, azurite and malachite, appears to be associated with aspects of the social identity of the deceased. First, application of cinnabar to the head appears to have been reserved mainly for males. Throughout history cinnabar was of special importance in many cultures for its bright red colour, resistance to fading over time and its hypnotic and sedative properties when heated^94–98^. The special value of cinnabar is also suggested for Çatalhöyük, where it was only present in a few burials and wall paintings^81,92,99^. Interestingly, the restricted presence of cinnabar to the frontal and temporal bones accompanied by phytoliths may suggest the once presence of a headband which might also have been worn during life^85,100^. Ethnic groups from Vanuatu consider headbands, regardless of colour, as valuable objects, only worn by men of high rank^101,102^, and in Neolithic and Bronze Age China cinnabar was exclusively linked to elite graves^103^. It is important to remain cautious with drawing such parallels, but the presence of cinnabar may suggest that these individuals had obtained a special status. Although a discussion of the type and transmission of social status at Neolithic Çatalhöyük is beyond the scope of this contribution, it is interesting to note that kinship studies in progress so far failed to show genetic relatedness between individuals bearing traces of cinnabar and between individuals buried in the same domestic structures^104–107^. This may suggest that in the Çatalhöyük community status was acquired rather than inherited. However, caution is required given the small sample size.

The second trend shown by the results is that blue and green burial associations were solely present in burials of females and subadults. Blue and green colours entered the burial record as early as the Late Natufian, from ca. 11,000 cal BC^108,109^. These colours have sometimes been associated with concepts of growth, fertility and ripeness, which are abstractions that could be related to the transition to agriculture^108,109^. In six cases at Çatalhöyük, green or blue pigment was associated with a bone object, interpreted as pigment applicators^90,110^ (Fig. 2d). The limited sample size precludes meaningful statistical comparisons for a more general interpretation, but this occurrence has significance for insight into practical aspects of application of colourants.

Evidence for pigment processing, pigment containers and application tools was found in the form of schist palettes, shell containers, a wooden bowl and animal bone applicators^85,90,110–112^. Fifteen shells (14 *Unio* sp. and 1 *Ostrea edulis*) out of a total of 29,395 shells (0.05%) recovered from various contexts at Çatalhöyük were categorised as ‘palettes’ or containers in which pigments had been mixed with a binder^85,113,114^. Eleven of those were recovered as burial associations (Table 1). The inner sides of these palettes showed traces of brush strokes in either cinnabar or ochre (Fig. 2e and Supplementary Table S5). Examination of the architectural use of colourants in combination with experimental studies have also shown that they were likely applied with (perishable) brushes onto dry plaster, after mixing the pigments with water^32,81,83,92^. Microchemical analyses of some of the paintings showed that the red paint consisted of a fine grained sediment of clay, calcite and haematite, together with embedded grains of red and colourless obsidian which may have had an effect on the optical properties of the artwork^92,115^.

When focussing on funerary evidence, only a few studies discuss the pigment application method. In a collective Natufian grave from Azraq, pigment was applied to crania as part of a secondary mortuary practices^24^. The Natufian site of Shubayqa in Jordan displayed child remains with ochre, suspected to have been stained from a burial container or wrapping^34^. In the Anatolian Early Neolithic preceramic sites of Körtik Tepe, Demirköy and Hasankeyf Höyük designs with lines were found on articulated elements^40–42^. These lines are suspected to have been transferred to the bones from painted matting or textiles as a result of soil pressure^40,41^, although it is also suggested that some bones may have been painted directly as part of a post-depositional treatment^40^. An early PPNB deposit at Dja’de el-Mughara revealed a subadult surrounded with phytoliths and red colourant, interpreted as a stained mat^25,30^. The results obtained in the present study on Neolithic Çatalhöyük show a significant difference in pigment application between adults and subadults with pigment exclusively on the cranium being more frequent in subadults. This may suggest that pigment was manually applied to the head of younger individuals, while adults could have been stained on the cranium and infracranial skeleton from exposure to matting or from sprinkling colourant. This possibility should be studied in more detail. In four cases ochre appeared to be more concentrated on one side of the skeleton (skeletons 21884, 22522, 32645, 32437), observations that were also reported at Körtik Tepe^40^. Skeleton 21884 showed clear indications that staining occurred on the uppermost side of the skeleton in the grave (Fig. 2f–h). This shows that ochre was applied to the left side of the deceased after being placed in the grave on the right side. The partial staining of the left femoral head confirms that the skeleton had been flexed and fleshed when the ochre was applied, leaving a large part of the femoral head unstained, being protected by the acetabulum and associated soft tissues (Fig. 2h). The abundant presence of phytoliths in this burial confirms the use of matting, although the archaeological evidence makes it difficult to reconstruct if the mat had been placed on top or around the body. Therefore, it is difficult to conclude if the matting was painted with ochre or if ochre was sprinkled on top of the deceased, before being wrapped in matting. However, there is a likely association between pigments and the use of some form of wrapping or covering, either as matting or basketry. Analysis of the entire dataset of all primary, primary disturbed and secondary burials indicates that phytoliths were observed in just over 5% of burials^90^, while 67% of the sub-sample of skeletons with direct pigment traces showed evidence of some sort of wrapping or covering.

The presence phytoliths in combination with the presence of cinnabar on the frontal and temporal bones of crania, indicates that a headband could have been worn. However, it remains unclear if the deceased wore a headband stained with cinnabar, or a headband over a stripe of cinnabar applied to the skin. The fact that the observed phytoliths were unstained (Fig. 2c), does not necessarily exclude one of these options^85^. With regard to the timing of application, the stripes have most often been observed on individuals from primary depositions, which suggests that cinnabar was put on a fleshed head and not on dry skeletal remains. Over the years, both soft tissue of the deceased and organic matter of the headband degraded, leaving a coloured stripe on the cranium (Fig. 2a,b).

**Figure 7:**
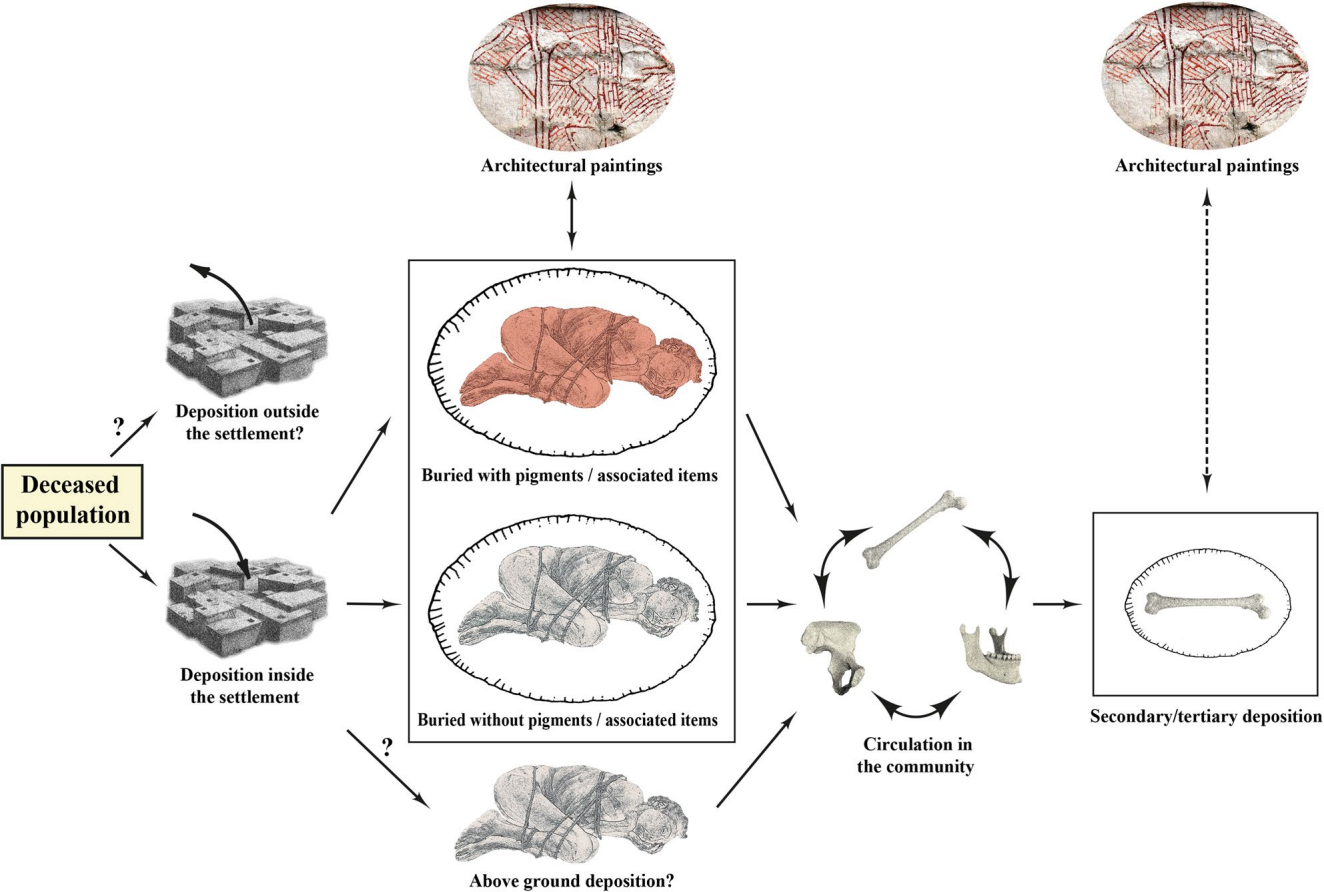
Hypothesised selection process. Deposition of some individuals outside the settlement cannot be completely excluded. The deceased, deposited inside the settlement, were selected to be buried with or without pigments and associated items. There was a tendency for these primary burials to be accompanied by architectural paintings. Other individuals, either as complete bodies or loose skeletal elements, remained in the community. These circulating skeletal elements were eventually deposited in secondary or tertiary deposition contexts, which may also have been linked to the creation of architectural paintings in an indirect way (dotted line), because not all tertiary depositions were associated with the occupation of the house only. Figure generated with Adobe illustrator 23.0.6 (http://www.adobe.com/fr/products/illustrator.html).

An important result from the joint analysis of mortuary and architectural data is the association between primary and tertiary depositions, and number of painted layers in the buildings. These connections have been previously suggested^68^, but were not statistically tested to date. The combination of these data suggest the following selection process (Fig. 7): (1) While previous population estimates of the size of the Çatalhöyük community^116^ might be overestimated^117^, the skeletal assemblage is consistent with an attritional mortality profile^77,78^. Nevertheless, the deposition of some individuals outside the settlement cannot be excluded based on other Neolithic Near Eastern sites where it has been suggested that not all individuals were buried inside the settlement^118–120^. (2) The results from this study show that primary burials, regardless of pigment presence, tend to be accompanied by architectural paintings. Some community members were selected to be buried with pigments and/or associated items but the majority did not receive this special treatment^77,79,90^. (3) A final selection included individuals, either as complete bodies or disarticulated skeletal elements, who remained in the community as a result of delayed burial practices or by re-opening burials^77,79,121^. These circulating skeletal elements were eventually deposited in secondary or tertiary deposition contexts, which may also have been linked to the creation of architectural paintings.

The selection process detailed in Fig. 7 might reflect a form of early social differentiation, as suggested before in the literature^58,59^. Sex and/or age-at-death alone did not influence selection for mortuary treatment^77,121,122^ and genetic relatedness did not play a major role either^104,106,107^. More research is necessary to understand which factors motivated the choices (e.g. vertical vs. horizontal social differentiation).

The results of this study also show that tertiary depositions might be more important than previously thought, irrespective of skeletal element involved. Only recently, the tertiary assemblage from Çatalhöyük received some attention^77^. The Near Eastern Epipalaeolithic and Neolithic literature mostly focuses on the circulation and secondary treatments of crania and entire skulls rather than other isolated infracranial elements in non-burial contexts^36,59,121,123–127^. The analysis of skeletal remains in less typical depositional locations deserves further consideration, as do reflections on dynamic mortuary sequences as opposed to graves as static features.

The results could be indicative of the construction of ‘social memory’, as has been argued for other Epipalaeolithic and Neolithic communities in the Near-East^59,60,128–132^. According to socio-cultural anthropologists, collective memory is handed down from generation to generation through repetition of past actions and by direct object-to-memory association^133^. Intramural burials may have been a part of processes of memory retention with each interment contributing to communal memory by keeping the deceased close to the daily rhythm of repeated household activities^65^. In Çatalhöyük the buildings show repetitive patterning and repetition of practical routines^60,90,92,128^. The creation of architectural uses of colourants associated with burial rites fits well in this scenario. The circulation and deposition of human remains can therefore be linked to object-to-memory association^60,128^.

It appears that forms of commemoration at Çatalhöyük changed over time. This is suggested by a change in the relative proportion of primary versus secondary/tertiary depositions, with an increase of secondary/tertiary depositions over time (Fig. 4a)^77^. In addition, the frequency of individuals deposited with pigments decreases from the earliest to the latest occupation periods (Fig. 4b). Similarly, there is a decline in architectural pigment use around 6500 cal BC, in combination with changes in spatial location and distribution. These results correspond with former suggestions that inhabitants from the Early levels of Çatalhöyük may have been held together by collective memory, while the community in the Late and Final periods was increasingly less dependent on cohesive ritual ties^75^. These changes, at least for the specific society under study, may also have been linked to a shift to different means of symbolic expression, as suggested by the parallel increase of other, more portable forms of visual culture such as pottery^134^ and stamp seals^135^. These patterns might also be linked to a greater emphasis on the concept of ownership which is associated with early farming^136^. The diachronic changes are consistent with and provide support for previous observations at Çatalhöyük regarding increased productivity, increased social autonomy of single households, and the shift towards a more dispersed settlement pattern, reduced ritual ties and dispersal of the population around 6500 cal BC^73,75,76,137^.

## Conclusions

Human remains, pigments and architectural use of colourants from Neolithic Çatalhöyük provide new insights into pigment use by this community, by showing the internal dynamics of this particular society and contributing to the interpretation of mortuary practices in Neolithic Anatolia. The results reveal a correlation between the use of pigments in funerary and architectural contexts. They suggests that dynamic mortuary actions were integrated into a variety of social practices consisting of specific selection processes largely independent of sex and age-at-death of the deceased. This study also highlights the significance of tertiary depositions, in addition to those related to the practice of cranial retrieval. Future excavations, aDNA analyses, increased interdisciplinary research and (re)-examination of tertiary depositions may provide further information about early social complexity of this Neolithic farming community, its symbolism and ideology. Undoubtedly other colourful materials of an organic nature would also have been part of the colour palette, and the current data likely provide an incomplete glimpse of the actual frequency of practices employing colour at Çatalhöyük. In the future, a consistent and systematic analysis of all pigments based on the same recording system at a number of contemporary sites would be useful to permit inter-site, regional and interregional comparisons. This should increase considerations specifically focused on mortuary pigment use in Neolithic Anatolia and, ultimately, in the Neolithic Near East.

## Materials and methods

### Çatalhöyük deposition categories

In total, a minimum of 816 individuals were excavated from stratified Neolithic contexts during the Hodder excavations (1993–2017). Each burial was assigned to one of several deposition categories (*primary undisturbed, primary disturbed, secondary, tertiary and unknown* burials). A detailed description of each category is reported elsewhere^77,78,122^. In brief, *primary undisturbed* depositions include articulated skeletons not disturbed by subsequent Neolithic activity (n = 286). *Primary disturbed* depositions include articulated skeletons found in their original/initial location of burial but were disturbed by subsequent Neolithic activity (n = 185). *Secondary burials* are represented by disarticulated or partially articulated skeletons retrieved from a primary burial location and intentionally deposited in another during the Neolithic (n = 96). The category of *tertiary depositions* consists of isolated, disarticulated or partially articulated skeletal elements found outside typical burial contexts, in internal spaces (e.g. construction, occupation and abandonment layers) as well as external spaces (e.g. middens, open areas) (n = 174). Lastly, the *unknown* category includes skeletal remains for which the original deposition could not be determined due to disturbance (e.g. soil erosion, animal burrowing) (n = 75).

### Biological profiles

Skeletal remains were studied using standard macroscopic biological anthropological methods. For adults, age-at-death was assessed using morphological changes of the auricular surface^138^ and pubic symphysis^139^. For non-adult individuals, age-at-death was estimated based on timing of epiphyseal fusion^140^, dental development and mineralisation^141^. The individuals were grouped in the following age cohorts: old adults (above 50 years of age-at-death), middle adults (35–49 years of age-at-death), young adults (20–34 years of age-at-death), adolescents (12–20 years of age-at-death), children (3–12 years of age-at-death), infants (between 2 months and 3 years of age-at-death), neonates (from birth to 2 months of age-at-death) and pre-term fetuses (prenates). Sex determination was carried out based on sexually-diagnostic morphological characteristics of the pelvis, cranium and mandible^142–145^. Individuals were categorised as female (*F*), possible female (*F?*), indeterminate (*indet*), possible male (*M?*), male (*M*) and too young to determine (i.e. < 18–20 years old) (*tytd*).

### Pigments

All burials (primary and secondary) that showed presence of pigment were selected from the Çatalhöyük Project Database and the annual archive reports^146^. Each individual was further classified according to the relationship between pigments and skeletal remains in (1) skeletons with direct pigment traces and (2) individuals with burial associations that contained pigment (Table 1).

Intentionality was demonstrated based on analyses of reference sediment samples, relative location, size, shape and use wear analysis of the objects with pigment. Microscopic analyses have shown that yellow residues may have been misidentified as pigment while being botanical remains or discolouration of the sediment^147^. In addition, not all ‘yellow pigment’ appeared to have been sampled, so it could not be verified geochemically. Therefore, it was decided to exclude possible yellow ochre from the database.

Skeletal remains and associated items, documented to have pigments and when able to be traced in the archived collection, were analysed using portable X-ray fluorescence (PXRF). The pigments were analysed using a portable SPECTRO xSORT X-ray fluorescence spectrometer (ED-XRF) from Ametek equipped with a silicon drift detector (SDD) and a low power W X-ray tube with an excitation source of 40 kV. Samples were positioned above a 7 mm diameter aperture and analysed over an acquisition time of 10 s. PXRF investigates only the elemental composition of samples. This means that characterisation of pigments was based on the presence of specific elements such as mercury (Hg) in cinnabar (HgS) (see Supplementary Fig. S1) and the presence of copper (Cu) in azurite (Cu_3_(CO_3_)_2_(OH)_2_) or malachite (Cu_2_CO_3_(OH)_2_) (see Supplementary Figs. S2 and S3). To confirm that these elements were not derived from the depositional environment, a reference sediment sample was analysed as a control (see Supplementary Figs. S1, S2, S3). The XRF results are presented in Supplementary Table S4.

Some pigments were exported to the Middle East Technical University (METU) for further analysis with X-ray Diffraction (XRD) (see Supplementary Table S4). Exports were limited to pigments from elaborate burials or unidentifiable pigments. XRD patterns were recorded on a Rigaku MiniFlex II X-ray diffractometer (wavelength of X-rays 0.154 nm Cu source, voltage 30 kV, filament emission 15 mA). Samples were scanned from 10 to 80° (2θ) using a 0.01° step width and a 1 s time count. The divergence slit was 0.3°. The powder patterns were converted to be evaluated using the DIFFRAC.EVA V3.1 software package.

### Architectural pigment use

Most of the buildings excavated to date yielded evidence of some form of colourants on walls of other surfaces, although with a great variability^32,81–83,92^. For the architectural use of colourants, information was gathered based on searching the main excavation database, followed by direct examination of painted plaster sequences through in situ examination and cross-sectional sampling for laboratory analysis. Cross-sectional sampling was undertaken to investigate painting frequency and relative temporal location within multi-layered plaster sequences reflecting the lifespan of a building. An entire cross-sectional plaster sequence was considered as a unit. Within the archaeological unit, the plastering events as well as painted layers were counted, resulting in a data category called ‘minimum number of painted layers’ (Fig. 5a). The database contained details of the context of each instance of colourant-bearing plaster, the minimum number of painted layers within the plaster sequence, the state of exposure and preservation, and, if possible, the type of motif (e.g. monochromatic red (Fig. 5b), geometric (Fig. 5c) or hand motif), and additional observations.

### Statistical analysis

Quantitative analyses were conducted to investigate relationships between pigment use and burial practices. All depositions examined in this study were recovered from stratified contexts within buildings where at least 15% of the spatial coverage was excavated^148^. This results in a sample of 66 buildings from the three main temporal groupings referred to as Early (n = 13), Middle (n = 31) and Late (n = 22) occupation periods. Due to the nature of the data, the analyses were carried out in two parts. First, the individual burials were examined to explore possible associations between pigment application and burial characteristics. In particular, the analysis focused on assessing if the occurrence of pigment staining on the cranium versus post-cranial elements varied by the age-at-death and sex of the individual. Here, either a Chi-Square Test or Fisher’s Exact Test was used depending on sample size. In order to maximize sample size and facilitate statistical comparison individuals were grouped in two categories only: adults (above 20 years age-at-death) and subadults (below 20 years age-at-death).

In the second part of the analysis on the association between the number of buried individuals and the number of painted layers in buildings, correlations between burial frequencies and the occurrence of painted layers were tested using Kendall’ Tau to accommodate the presence of outliers and the non-symmetrical data distribution commonly encountered among count data. Then, the association between burial frequency among different depositional categories (irrespective of the presence of pigment) and the occurrence of painted layers in buildings was explored by constructing a generalised linear model (GLM). Depending on model dispersion, either a Poisson or a negative binominal error distribution was used to accommodate the count data. The analysis was limited to buildings that were more fully excavated (≥ 75% excavated; n = 23) to minimise bias in the frequency of recovered burials due to insufficient excavation coverage. To construct the GLM, the number of painted layers in each building was set as the response variable, while the number of primary (undisturbed *plus* disturbed) and tertiary depositions were inputted as predictors. Both predictor variables were first transformed to achieve an approximately symmetrical distribution to fulfil the assumptions of linear modelling, then scaled and centred to facilitate the interpretation of the model coefficients. The model was checked for collinearity with variance inflation factor, over-/under-dispersion, and influential cases with Cook’s Distance and leverage. Secondary burials were excluded from the analysis due to their low numbers or absence in most of the 75% excavated buildings analysed here. For reasons of sample size, the tertiary deposition category included all internal contexts related to a specific building, including house construction, occupation, modification and closure/abandonment layers. The distribution of ≥ 75% excavated buildings by time period appeared uneven, with a bias toward the Middle occupation period (n = 15, which is 65% of buildings excavated ≥ 75%). For this reason, time period was not included in the GLM as a predictor variable, as its uneven data balance may have affected model stability.

All statistical comparisons were conducted using an alpha value of 0.05. All analyses are conducted using the R statistical software (R Core Team 2019). The R code in rMarkdown format and data required to reproduce the statistics and associated graphics in this study are provided as supplementary material (see Supplementary S6 and S7).

### Statements

All methods were carried out in accordance with relevant guidelines and regulations. No human or animal experiments were carried out for this study. Neolithic skeletal remains were analysed with consent of the Turkish authorities under the permit from the Ministry of Culture and Tourism, General-Directorate of Cultural Heritage and Museums, provided to the Çatalhöyük Research Project under the direction of Prof. Ian Hodder. All experimental protocols were approved by the University of Bordeaux and the University of Wollongong.

## Supplementary Information

Supplementary Information 1.

Supplementary Information 2.
