# Supplementary material for: New insights on commemoration of the dead through mortuary and architectural use of pigments at Neolithic Çatalhöyük, Turkey
Source: Sci Rep. 2022 Mar 8;12:4055. doi: 10.1038/s41598-022-07284-3 (PMC8904496; doi:10.1038/s41598-022-07284-3)

Supplementary online material

New insights on commemoration of the dead through mortuary and architectural use of pigments at Neolithic Çatalhöyük, Turkey

Schotsmans E.M.J.[1, 2, *], Busacca G.[3], Lin S.C.[2, 4], Vasić M.[3], Lingle A.M.[5], Veropoulidou R.[6], Mazzucato C.[7], Tibbetts B.[8], Haddow S.D.[9], Somel M.[10], Toksoy-Köksal F.[11], Knüsel C.J.[1], Milella M.[12]

[1] Laboratoire d'Anthropologie des Populations Passées et Présentes (PACEA), UMR 5199, Université de Bordeaux, Pessac, France ; [2] Centre for Archaeological Science, University of Wollongong, Wollongong, Australia; [3] Independent researcher; [4] ARC Centre of Excellence for Australian Biodiversity and Heritage, University of Wollongong, Wollongong, Australia; [5] School of History, Archaeology, and Religion, Cardiff University, Cardiff, United Kingdom; [6] Museum of Byzantine Culture, Hellenic Ministry of Culture and Sports, Thessaloniki, Greece; [7] Department of Anthropology, Stanford University, Stanford, USA; [8] Department of Archaeology, University of Exeter, Exeter, United Kingdom; [9] Department of Cross-Cultural and Regional Studies, University of Copenhagen, Copenhagen, Denmark; [10] Department of Biological Sciences, Middle East Technical University (METU), Ankara, Turkey; [11] Department of Geological Engineering, Middle East Technical University (METU), Ankara, Turkey; [12] Department of Physical Anthropology, Institute of Forensic Medicine, University of Bern, Bern, Switzerland; * Corresponding author

Table of contents

S1: PXRF spectra of a cinnabar stripe, cinnabar shell and reference sample p2

S2: PXRF spectra of dark azurite and reference sample p3

S3: PXRF spectra of light azurite and reference sample p4

S4: List of analysed pigment samples p5

S5: List of analysed shells from burial contexts p5

S6: R Markdown file used for the statistical tests and the Generalised Linear Model p6

S7: Online zipfile with R code in rMarkdown format and data required to reproduce statistics

Supplementary S1

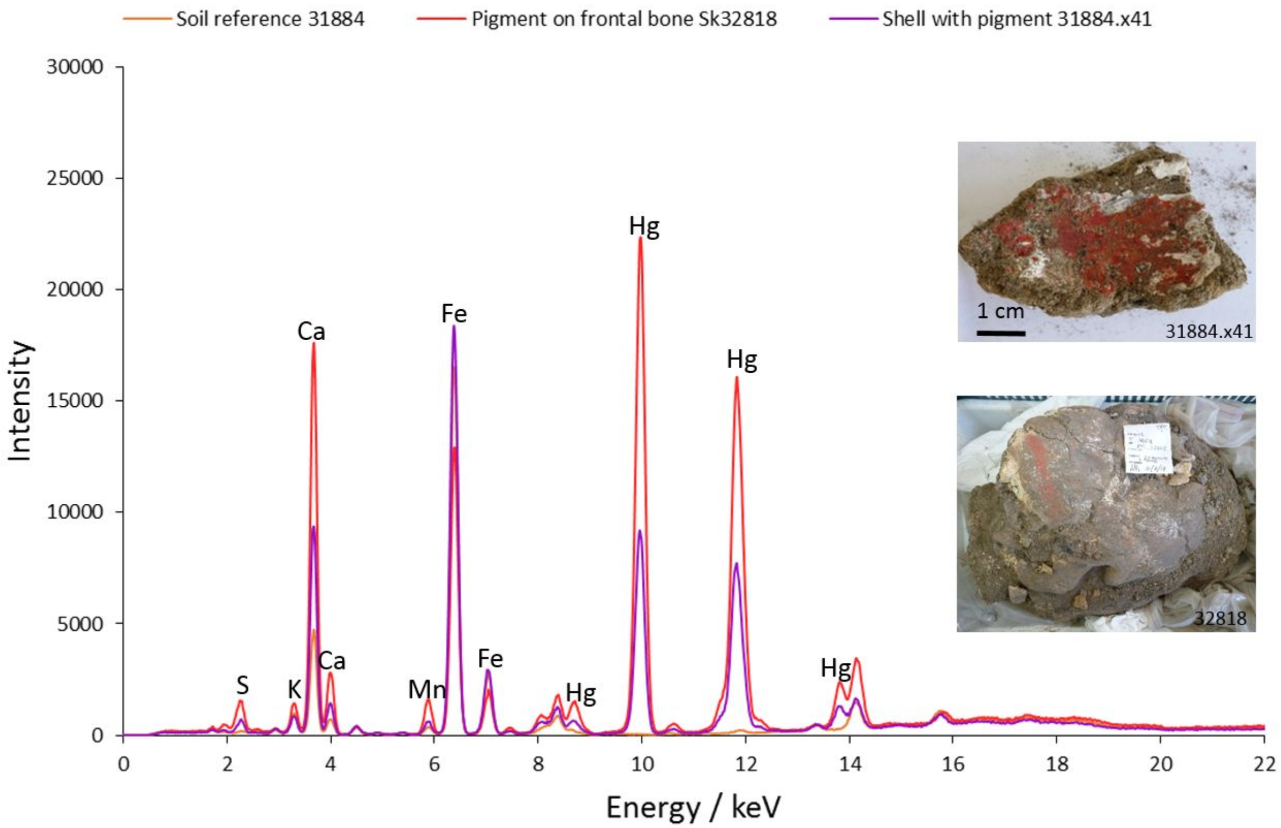

Figure S1: PXRF spectra of red pigment found on the frontal bone of skeleton 32818 (in red) and on shell 31884.x41 (*Unio* sp.) (in purple) recovered as burial association with the same individual. Both pigments were characterised as cinnabar (HgS) by the presence of sulphur (S) and mercury (Hg). The reference sediment sample (31884) from the burial fill (in orange) did not contain any of these elements. Spectra generated using Microsoft Office Excel 2016 (https://www.microsoft.com/en-us/microsoft-365/excel) and combined with images in Adobe illustrator 23.0.6 (http://www.adobe.com/fr/products/illustrator.html).

Supplementary S2

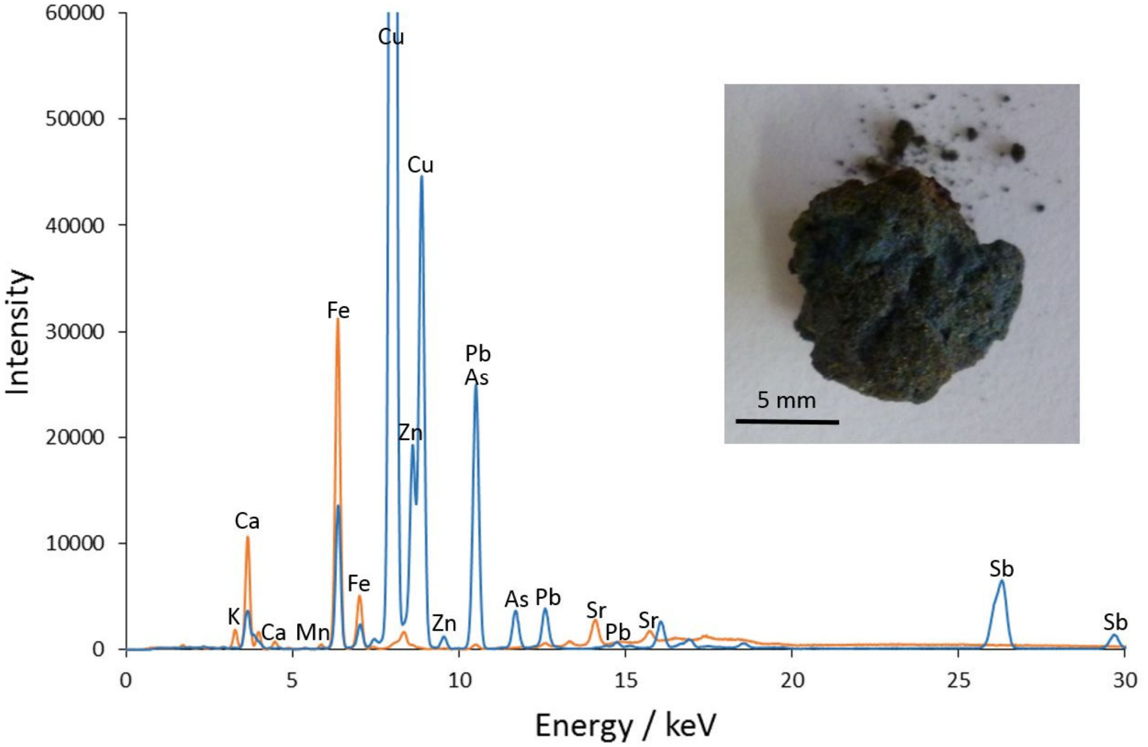

Figure S2: PXRF spectra of dark blue pigment (in blue) (30039.S9) found with skeleton 23126 and of a reference sediment sample (in orange) from the grave fill (30039.S2). The blue pigment was identified as azurite ($Cu_3(CO_3)_2(OH)_2$) of the copper arsenite group, indicated by the presence of copper (Cu), arsenic (As), antimony (Sb), lead (Pb) and zinc (Zn), and confirmed to be azurite with XRD. These elements are absent in sediment sample 30039.S2 which shows the presence common sediment elements potassium (K), calcium (Ca), iron (Fe) and strontium (Sr). Spectra generated using Microsoft Office Excel 2016 (https://www.microsoft.com/en-us/microsoft-365/excel) and combined with image in Adobe illustrator 23.0.6 (http://www.adobe.com/fr/products/illustrator.html).

Supplementary S3

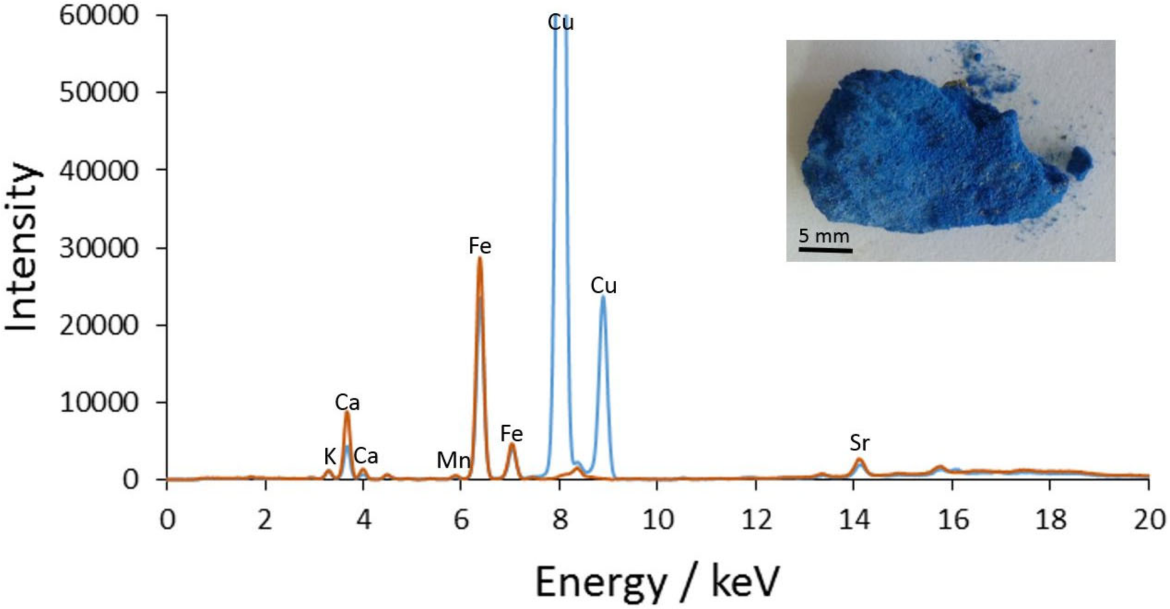

Figure S3: PXRF spectra of blue pigment (in blue) (31888_s8) found in a grave with several individuals in building 150 and of a reference sediment sample (in orange) from the grave fill (31888_s1). The blue pigment was identified as azurite ($Cu_3(CO_3)_2(OH)_2$) indicated by the presence of copper (Cu) and confirmed with XRD. These elements are absent in sediment sample 31888_s1. Its spectra shows the presence of common sediment elements potassium (K), calcium (Ca), manganese (Mn), iron (Fe) and strontium (Sr). Spectra generated using Microsoft Office Excel 2016 (https://www.microsoft.com/en-us/microsoft-365/excel) and combined with images in Adobe illustrator 23.0.6 (http://www.adobe.com/fr/products/illustrator.html).

Supplementary S4

Table S4: List of analysed pigment samples with main XRF results indicated by the presence of colourant-specific elements. Table generated using Microsoft Office Excel 2016 (https://www.microsoft.com/en-us/microsoft-365/excel).

Skeleton (unit) number	Sample unit number	Pigment notes	XRF	XRD	Presence of colourant specific elements with XRF				Pigment	Figure or table reference
					Fe	Hg	S	Cu		
32818	32818	Stripe frontal bone	✓			✓	✓		HgS	Fig. 2a, 2b, S3
11330	11330	On plastered cranium	✓		✓				Fe_2O_3	
17533	17533	Left side cranium	✓			✓	✓		HgS	
32741	32741	On occipital fragments and ribs	✓		✓				Fe_2O_3	
32762	32762	Cranium, right ulna, vertebral	✓		✓				Fe_2O_3	
32045	32045	Parietal bone	✓		✓				Fe_2O_3	
22196	22196.S10	Stripe on frontal bone	✓	✓		✓	✓		HgS	Fig. 2c
32330	32330.S2	Cranium	✓	✓		✓	✓		HgS	
30523	30523	Frontal bone	✓			✓	✓		HgS	
23805	23805	Frontal bone	✓			✓	✓		HgS	
30007	30007	Cranium and specks on cervical	✓			✓	✓		HgS	
30010	30010	Cranium	✓			✓	✓		HgS	
20685	20685	Temporal bone	✓			✓	✓		HgS	
8598	8598	Infracranial skeleton	✓		✓				Fe_2O_3	
22335	22335	Frontal bone	✓			✓	✓		HgS	
22335	22335	Cranium	✓		✓				Fe_2O_3	
4424	4424	Frontal bone	✓			✓	✓		HgS	
21884	21884	Whole skeleton (more left)	✓		✓				Fe_2O_3	Fig. 2f, 2g, 2h
23238	23238.S6	Pelvis, lower limbs	✓		✓				Fe_2O_3	
21817	21817.S5	Whole skeleton	✓		✓				Fe_2O_3	
21855	21855	Torso and upper limbs	✓		✓				Fe_2O_3	
22522	22522	Infracranial skeleton	✓		✓				Fe_2O_3	
22522	22522	Cranium	✓			✓	✓		HgS	
5177	5177	Temporal bone	✓			✓	✓		HgS	
32645	32645	Whole skeleton (more right)	✓		✓				Fe_2O_3	
32646	32646	Whole skeleton	✓		✓				Fe_2O_3	
32437	32437	Infracranial skeleton	✓		✓				Fe_2O_3	
32437	32437.S1	Cranium	✓	✓		✓	✓		HgS	
23237	23237	Whole skeleton	✓		✓				Fe_2O_3	
32818	31884.X41	Red pigment on shell	✓			✓	✓		HgS	Fig. 2a, S3, S5
Several	31888.S12	Blue lump	✓	✓				✓	$Cu_3(CO_3)_2(OH)_2$	Fig. S2
2842	2841.X2	Red pigment on shell	✓			✓	✓		HgS	S5
22196	22194.S10.X6	Red pigment on shell	✓	✓		✓	✓		HgS	Fig. 2e, S5
23126	30039.S14	Red lump of pigment and red	✓	✓	✓				Fe_2O_3	
23126	30039.S15.S9	Blue lump	✓	✓				✓	$Cu_3(CO_3)_2(OH)_2$	Fig. S1
21672	21634	Green pigment on bone	✓	✓				✓	$(Cu_2CO_3(OH)_2)$	
17939	17939.X1	Red pigment on shell	✓			✓	✓		HgS	S5
30199	22065.X3	Red pigment on shell	✓			✓	✓		HgS	S5
17457	17457.X1	Red pigment on shell	✓			✓	✓		HgS	S5
17457	17457.X6	Red pigment on shell	✓		✓				Fe_2O_3	S5
22516	22515	Lump of red pigment	✓			✓	✓		HgS	

Supplementary S5

Table S5: List of studied shells from burial contexts (R. Veropoulidou) with PXRF results (E. Schotsmans). Table generated using Microsoft Office Excel 2016 (https://www.microsoft.com/en-us/microsoft-365/excel).

Period	Level	Unit	Find	Building	Skeleton	Species	Type	Use wear	PXRF results
Late	TP-M	31884	X41	**150**	32818	*Unio (mancus)*	Container/palette		Cinnabar
Middle	South ?M	2841	X2	**50**	2842	*Unio (mancus)*	Container/palette		Cinnabar
Middle	North F	22194	X6	**5**	22196	*Unio (mancus)*	Container/palette		Cinnabar
Middle	North G	17939	X1	**49**	17939	*Unio (mancus)*	Palette	Scratches, furrows, concentric striations due to drilling (internal surface); perforation	Cinnabar
Middle	North G	22065	X3	**77**	30199	*Unio (mancus)*	Container/palette		Cinnabar
Middle	North G	17457	X4	**49**	17457	*Unio (mancus)*	Container/palette	Brushing (internal, above pallial line)	Cinnabar
Middle	North G	17457	X6	**49**	17457	*Unio (mancus)*	Container/palette		Ochre

Supplementary S6

S6: R Markdown file used for the statistical tests and the Generalised Linear Model (S. Lin).

All images in S6 were generated with R x64 v4.1.0 (https://cran.r-project.org/bin/windows/base/old/4.1.0/).

R packages used include:

- *ggplot2* (Wickham, H. 2016. ggplot2: Elegant Graphics for Data Analysis. Springer-Verlag, New York.)
- *ggpubr* (Kassambara, A. 2017. ggpubr: "ggplot2" Based Publication Ready Plots. R package version 0.1.6. https://cran.r-project.org/package=ggpubr.)
- *scales* (Wickham, H., and Seidel, D. 2019. scales: Scale functions for visualization. R package version 1.1.0. http://cran.r-project.org/package=scales.)
- *msme* (Hilbe, J., and Robinson, A. 2018. msme: Functions and Datasets for "Methods of Statistical Model Estimation". R package version 0.5.3. https://CRAN.R-project.org/package=msme)
- *car* (Fox, J., and Weisberg, S. 2019. An {R} Companion to Applied Regression, Third Edition. Thousand Oaks CA: Sage. URL: https://socialsciences.mcmaster.ca/jfox/Books/Companion/)
- *dplyr* (Wickham, H., François, R., Henry, L., and Müller, K. 2021. dplyr: A Grammar of Data Manipulation. R package version 1.0.7. https://CRAN.R-project.org/package=dplyr)

Fisher's exact test: Pigment on cranium only vs. cranium and post-cranial elements across age groups

```
##
##                         Adult Subadult
##   Cranium + Post-cranium    12        2
##   Cranium-only               6       10

##
##  Fisher's Exact Test for Count Data
##
## data:  age.test.matrix
## p-value = 0.01061
## alternative hypothesis: true odds ratio is not equal to 1
## 95 percent confidence interval:
##    1.341165 112.520625
## sample estimates:
## odds ratio
##   9.161408
```

Fisher's exact test: Pigment direct application vs. by association across time period

```
##
##            Early Middle Late
##   Associated     3     19    4
##   Direct        18     14    4

##
##  Fisher's Exact Test for Count Data
##
## data:  period.test.matrix
## p-value = 0.003679
## alternative hypothesis: two.sided
```

Fisher's exact test: Number of painted layers across time period

```
##
##                   Early Middle Late
##   No painted layers     5      9   15
##   Painted layers        8     22    7

##
##  Fisher's Exact Test for Count Data
##
## data:  period.test.matrix
## p-value = 0.01766
## alternative hypothesis: two.sided
```

Cross-tab summary of the number of burials with/without pigment and their association with/without painted layers

```
## # A tibble: 2 x 3
##   Painted pigment no_pigment
##   <chr>     <int>      <int>
## 1 N             0         22
## 2 Y            37        339
```

Kendall's tau correlation test: Number of burials with vs. without pigments among the buildings

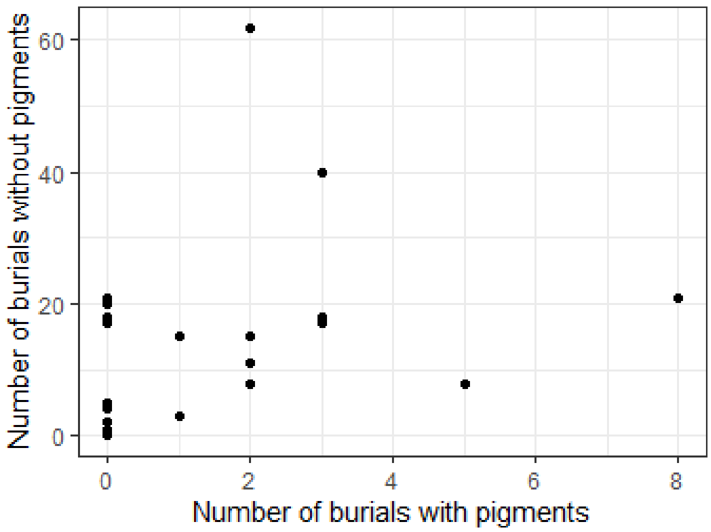


```
## Warning in
## cor.test.default(house_data_excavated$Num_burial_pigments_ALL_recoded, : Cannot
## compute exact p-value with ties

##
##  Kendall's rank correlation tau
##
## data:  house_data_excavated$Num_burial_pigments_ALL_recoded and house_data_excavated$Num_tot
al_individual_ALLCAT - house_data_excavated$Num_burial_pigments_ALL_recoded
## z = 2.2125, p-value = 0.02693
## alternative hypothesis: true tau is not equal to 0
## sample estimates:
##       tau
## 0.3659447
```

Kendall's tau correlation test: Number of primary burials with vs. tertiary burials among the buildings

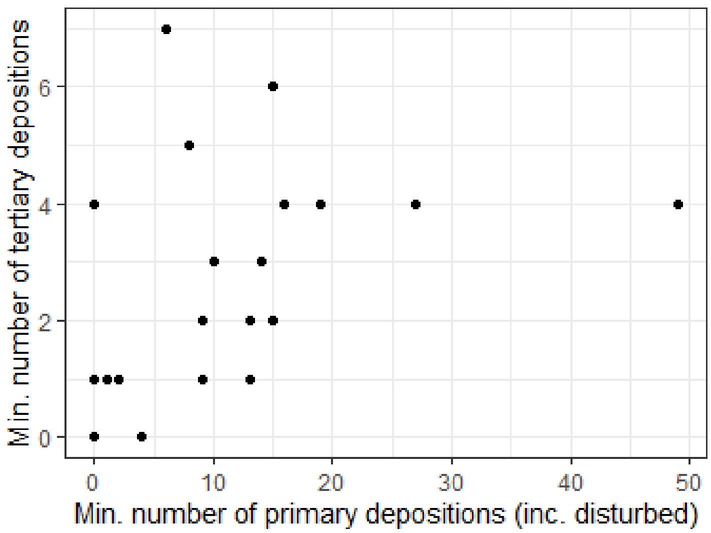

```
## Warning in cor.test.default(house_data_excavated$Num_primary_all,
## house_data_excavated$Num_tertiary, : Cannot compute exact p-value with ties
```

```
##
##  Kendall's rank correlation tau
##
## data:  house_data_excavated$Num_primary_all and house_data_excavated$Num_tertiary
## z = 2.6287, p-value = 0.008571
## alternative hypothesis: true tau is not equal to 0
## sample estimates:
##       tau
## 0.4239852
```

Figure 6a: Generalised linear model summarising the relationship of the number of primary and tertiary burials on the number of painted layers

```
##
## Call:
## glm.nb(formula = Num_painted_layers ~ scale(Num_primary_all_tr) +
##     scale(Num_tertiary_tr), data = house_data_subset, init.theta = 1.797598618,
##     link = log)
##
## Deviance Residuals:
##      Min        1Q    Median        3Q       Max
## -2.43474  -0.91988  -0.09169   0.55835   1.61937
##
## Coefficients:
##                           Estimate Std. Error z value Pr(>|z|)
## (Intercept)                 1.9153     0.1895  10.109  < 2e-16 ***
## scale(Num_primary_all_tr)   0.7986     0.2436   3.278  0.00105 **
## scale(Num_tertiary_tr)      0.4753     0.2359   2.015  0.04387 *
## ---
## Signif. codes:  0 '***' 0.001 '**' 0.01 '*' 0.05 '.' 0.1 ' ' 1
##
## (Dispersion parameter for Negative Binomial(1.7976) family taken to be 1)
##
##     Null deviance: 54.519  on 22  degrees of freedom
## Residual deviance: 26.147  on 20  degrees of freedom
## AIC: 146.09
##
## Number of Fisher Scoring iterations: 1
##
##
##               Theta:  1.798
##           Std. Err.:  0.712
##
##  2 x log-likelihood:  -138.091
```

Figure 6b: The relationship between primary (left panel)/tertiary (right panel) burial depositions and the minimum number of painted wall layers associated with the burials. The two black solid lines depict the modelled effect of primary and tertiary burials on the painted layers; the grey area represents the 95% confidence interval of the mean predicted values. In the left panel, note that there appears to be an outlier with an exceptionally high number of primary burials (n=49). However, the removal of this case has minimal influence on the resulting linear model due to the negative binomial error distribution used here. Figures generated with R x64 v4.1.0 (https://cran.r-project.org/bin/windows/base/old/4.1.0/).

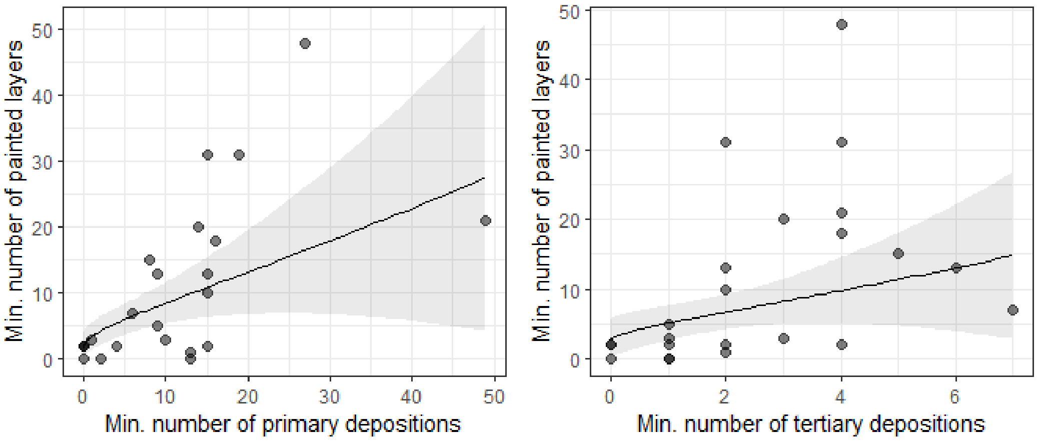

Supplement: Supplementary file 2 — Supplementary Information 2. [file 41598_2022_7284_MOESM2_ESM.pdf]
